# Comparison and Analysis of Several Clustering Algorithms for Pavement Crack Segmentation Guided by Computational Intelligence

**DOI:** 10.1155/2022/8965842

**Published:** 2022-09-03

**Authors:** Dan Wang, Zaijun Zhang, Jincheng Zhou, Benfei Zhang, Mingjiang Li

**Affiliations:** ^1^School of Mathematics and Statistics, Qiannan Normal University for Nationalities, Duyun 558000, China; ^2^Key Laboratory of Complex Systems and Intelligent Optimization of Guizhou, Duyun 558000, China; ^3^Key Laboratory of Complex Systems and Intelligent Optimization of Qiannan, Duyun 558000, China; ^4^School of Computer and Information, Qiannan Normal University for Nationalities, Duyun 558000, China

## Abstract

Cracks are one of the most common types of imperfections that can be found in concrete pavement, and they have a significant influence on the structural strength. The purpose of this study is to investigate the performance differences of various spatial clustering algorithms for pavement crack segmentation and to provide some reference for the work that is being done to maintain pavement currently. This is done by comparing and analyzing the performance of complex crack photos in different settings. For the purpose of evaluating how well the comparison method works, the indices of evaluation of NMI and RI have been selected. The experiment also includes a detailed analysis and comparison of the noisy photographs. According to the results of the experiments, the segmentation effect of these cluster algorithms is significantly worse after adding Gaussian noise; based on the NMI value, the mean-shift clustering algorithm has the best de-noise effect, whereas the performance of some clustering algorithms significantly decreases after adding noise.

## 1. Introduction

Pavement crack detection methods that are efficient and reasonable must be used to evaluate the degree of pavement damage, and appropriate maintenance measures must be taken in order to achieve the goal of objectively detecting pavement damage and ensuring the safety, efficiency, and convenience of transportation on pavement [1]. In order to achieve this goal, pavement damage must first be detected. In recent years, there has also been some progress made in the research of pavement crack detection; however, in the actual operation process, manual visual inspection is still used for the purpose of crack detection. Manual detection has been unable to meet the rapidly developing needs of highway maintenance, and the detection speed of cracks has not been able to keep up with the requirements of pavement development [2]. Only relying on manual detection of pavement cracks is not an accurate enough method because it will lead to issues such as missed detection and false detection, both of which will disrupt traffic and are unable to guarantee personal safety. Even if a large number of people are sent out to inspect and evaluate the pavement of the highway, it will still take a significant amount of time to compile statistics based on the data that has been collected. As a result, it is of the utmost importance to conduct cracks in the pavement in a prompt and efficient manner [3–5].

The most common types of pavement cracks are transverse cracks that run in the same direction as the lane line, longitudinal cracks that run perpendicular to the lane line, block cracks and network cracks generated by transverse and longitudinal staggered, and so on [6]. The low contrast between the crack area and the background, the interference caused by the background, and the uneven damage degree of the cracks are the three factors that make it the most difficult to detect and identify cracks. Inspections were performed manually and visually for the most part in the traditional method. There will be a great deal of difficulty, as evidenced by a single method of detection, a low detection efficiency, a lengthy cycle, and a high risk. The proliferation of computer technology has led to the development of an increasing number of automatic detection methods. Automatic detection technology has significantly improved in terms of both its efficiency and accuracy in comparison with manual detection, which eliminates the need for human intervention wherever possible. Image processing is typically what is utilized when looking for cracks [7–9].

The advantages of high precision, high efficiency, and strong robustness are all possessed by modern image processing technology, which is analogous to the visual system of the human brain. Information about crack features can be extracted after various features, including the texture and geometry of cracks, which have been analyzed [10]. Image enhancement, feature extraction, and object segmentation are all components of the overarching process that comprises detection methods in image processing [11, 12]. The detection of crack defects using image processing techniques has been the subject of a significant amount of research activity in recent years, both domestically and internationally. Cracks were found by Oliveira et al. by analyzing the light and dark changes of box-borders and the distribution of dark pixels, but this method is susceptible to noise because of its reliance on light and dark changes. In order to achieve crack segmentation, they searched for the valley that was located near the highest peak in the gray histogram as a threshold. In order to improve the limitations of the conditional texture anisotropy (CTA) directional detection, Schmugge et al. proposed a free-form anisotropy (FFA) algorithm to detect pavement cracks. Using this algorithm, the authors sought out the path with the least amount of weight in each direction using an iterative search algorithm. However, due to the high amount of computational work required, this algorithm is not appropriate for use in real-time detection. In order to remove noise from crack images, Yan et al. utilized a variety of fusion and recombination filtering methods. Using this method, it is possible to remove various types of noise; however, it is very easy to lose information regarding cracks. Yang et al. first performed curve fitting on the pixels in the segmented image and then used the SVM model to achieve classification. For SVM training, the authors extracted the grayscale histograms of the local crack area and the background area. This method selected a smaller number of feature parameters, which resulted in a poor recognition accuracy. An automatic algorithm for the classification of cracks was suggested by Lettsome et al. After the crack image has been vectorized, the geometric features are separated, and the linear cracks are classified according to the slope and width. This allows the type and degree of crack damage to be determined, allowing for the accurate and automatic extraction of crack information. Lettsome et al. developed an improved pavement image segmentation algorithm, which extracts the statistical data, structure, and shape of pavement cracks in image sub-patches to construct crack-feature vectors and identifies the categories of sub-patches through sparse representation, so as to improve the accuracy of pavement crack recognition. This was done in order to improve the quality of the pavement crack recognition process. A method for detecting cracks in asphalt pavement based on its spatial aggregation feature has been proposed by one researcher. This method takes into consideration the spatial distribution characteristics, grayscale characteristics, and geometric characteristics of cracks. An adaptive threshold method was proposed by Liang et al., which is capable of realizing the extraction of small cracks. J. Knig et al. were successful in achieving their goal of detecting pavement cracks by combining the ICS-LBP method. This method is superior to the method of directly using gray value features because it can achieve real-time crack detection. Even though the methods of image processing have made some headway in the detection of pavement cracks, they are all based on the texture and geometric features of the surface. They are able to produce satisfactory results when dealing with a limited number of crack images. In spite of this, when confronted with a large number of complex crack images, the parameters of the texture geometry need to be adjusted in order to obtain satisfactory results, which cannot adapt to a greater variety of crack images [13–15].

With the advancement of technology, the use of image processing technology to detect pavement cracks has attracted an increasing amount of attention from scientific research institutions. As a result, many pavement detection systems have been developed, such as road crack, which is a pavement detection system that was developed in Australia. In accordance with the complexity of block cracks and network cracks, these two types of cracks are intertwined with horizontal and vertical cracks, and they develop into pavement cracks, which are not pavement cracks that appear early on in the process. As a result, the adaptability of the standard algorithm for crack segmentation is insufficient. In this paper, we will implement these mature clustering algorithms that are applied in engineering, and through the performance comparison of various algorithms, we will provide new ideas for pavement crack detection. In addition, the results of the tests can provide a decision-making basis for the work of maintaining the pavement, thereby lowering the costs of maintenance, improving economic benefits, ensuring driving safety, and developing a positive environment for infrastructure.

## 2. Related Works

### 2.1. Noise Model of Crack Image

**Figure 1:**
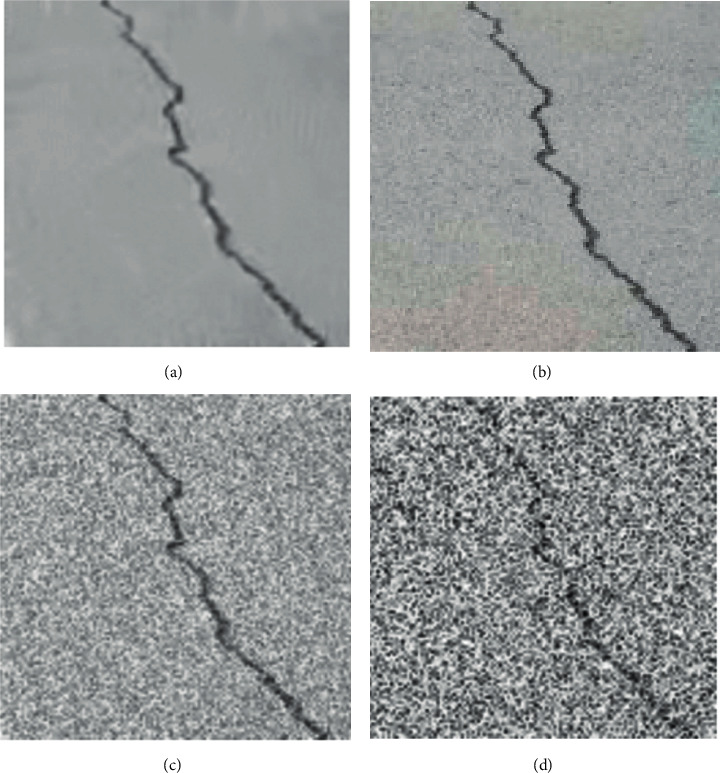
Noisy crack image. (a) RAW image; (b) noisy image with zero mean and 5 variance; (c) noisy image with zero mean and 10 variance; (d) noisy image with zero mean and 30 variance.

Noise, as we are all aware, is a random phenomenon that can only be characterized statistically. There are various types of noise, such as additive noise and multiplicative noise, among others. The statistical distributions of various noises, as well as the effects that those noises have on images, are each unique [16, 17]. Complex noise types can be seen in pavement images, with additive noise being the primary type. The noise model is presented in the following format for ease of analysis:

(1) $$\begin{array}{c} f\left(x\right)=x+n\text{,} \end{array}$$

where *f*(*x*) is the observed value; and *x* and *n* are the true value and the noise matrix, respectively. Gaussian noise is the most common additive noise.  Figure 1 shows the noisy crack image with different noises. For example, Figure 1(d) shows the image with zero mean and 30 variance. It can be seen that the noise obviously interferes with the crack and some details have been lost.

In general, the topological structure of a pavement fracture looks like a tree, while in specific areas it has a linear appearance. There are a number of very fine cracks, and the contrast between them varies quite a bit. In general, there are two types of crack images, which are known as crack backgrounds and false-crack backgrounds. One Rayleigh distribution and two normal distributions were chosen to simulate the statistical distribution of the crack background; the high gray area represents the fracture target, and a normal distribution was selected because it fits the statistical distribution of the crack image. The statistical results show that the probability of medium and low gray areas is designated as the crack background. One way to explain the statistical model is as follows:

(2) $$\begin{array}{c} f\left(x_{i}\right)=\omega_{4}f_{4}\left(x_{i}\right)+\sum_{l=1}^{3}\omega_{l}f_{l}\left(x_{l}\right)\text{,} \end{array}$$

where *ω*_*l*_(1 ≤ *l* ≤ 4) is weight coefficient; the constraints are ∑_*l*=1_^4^*ω*_*l*_=1;*f*_4_(*x*_*i*_) is the density function of Rayleigh distribution with parameter *γ*; and *f*_*l*_(*x*_*l*_)(1 ≤ *l* ≤ 3) is a normal distribution density function with mean and variance {*u*_*l*_, *σ*_*l*_^2^}. Therefore, the parameter set of crack image statistical distribution model is {*ω*_*l*_, *γ*, *u*_*l*_, *δ*_*l*_^2^}，*l*=1,2,3,4. In the practical application of engineering crack detection, the shape and structure of cracks are complex and random. The contrast of cracks in different positions is large, and there are a large number of peripheral cracks and fine cracks. These characteristics increase the difficulty of crack segmentation and seriously affect the accuracy of crack segmentation. Therefore, the traditional single grayscale feature of crack cannot effectively segment the crack, so the influence of crack characteristics and noise on the crack segmentation algorithm must be eliminated by introducing additional information. Therefore, image enhancement is used to eliminate the influence of noise and interference in this paper.

### 2.2. Crack Image Enhancement

**Figure 2:**
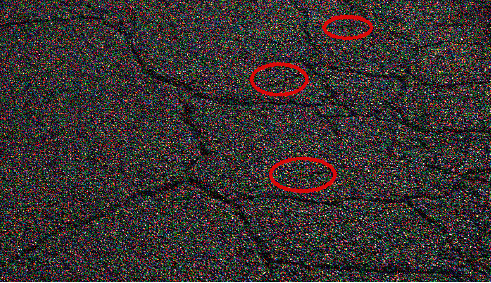
Comparison between cracks and noncracks.

The intensity of the light and the angle at which the photograph was taken caused the surface of the fallen concrete to produce shadows that resembled crack structures. This occurred during the process of acquiring the photograph. The false detection rate of cracks is increased as a result of these noncracks. It is challenging to differentiate between cracks and noncracks using only two-dimensional information because there is insufficient information regarding the depth of the three dimensions. The image of the crack that was collected in the real scene can be seen in Figure 2. The effect of the lighting on the image results in a lack of contrast, and the uneven pavement surface results in an uneven background area, both of which contribute to the lack of contrast between the cracks in the pavement. In addition, the red areas that can be seen in the image are not cracks but rather road scratches. However, the gradient characteristics of cracks are more readily apparent than those of false cracks, and appropriate filtering methods can be utilized to reduce the interference caused by false cracks.

Mean filtering, median image filtering, Gaussian image filtering, and nonlocal mean image filtering have emerged in recent years as the primary image filtering methods. The mean filter is the simplest, but it significantly reduces the prominence of edge features; the salt-and-pepper noise can be effectively removed by the median filter, but the crack image, which contains the vast majority of speckle noise, is not an appropriate candidate for this filter; the Gaussian filter has a beneficial effect on smoothing, and its overall performance is superior to that of the mean filter and the median filter; nonlocal mean filtering has a noise reduction effect that is comparable to that of Gaussian filtering. It also has the ability to preserve edge characteristics, despite the fact that the calculation is more complicated and the processing time is longer. In order to get rid of image noise and smooth out the gradient characteristics of false cracks, a two-dimensional Gauss low-pass filter has been chosen. This decision was made after taking into account the processing speed and effect in their entirety. The expression of filter can be shown like this:

(3) $$\begin{array}{c} G\left(u,\upsilon \right)=\frac{1}{2\pi \sigma }e^{-u^{2}+\upsilon^{2}/2\sigma^{2}}\text{,} \end{array}$$

where *σ*=1, and the sum of squares of *u* and *υ* represents the distance from the pixel (*u*, *v*) to the center.

The crack edge characteristics will be softened to some amount after image de-noising, and some crack details will be lost. As a result, it is required to improve the crack structure as well as the contrast of the crack. The Frangi filter, which is based on the Hessian matrix, is used to extract the linear structure in the image and suppress background clutter, which has a noticeable enhancing effect on the cracks. To calculate the gradient change rate of pixels, the Hessian matrix is built of second-order partial derivatives. The relationship between the eigenvalues *λ*_1_ and *λ*_2_ reflects the structure of the image. The Frangi filter detects the linear structure in the image by using the relationship between the eigenvalues *λ*_1_ and *λ*_2_ of the Hessian matrix and defines the eigenvalue linear parameters *R*_*b*_ and *S* to construct an enhancement filter function, which can distinguish the background and the target. According to the literature [8], these linear parameters can be written as follows:

(4) $$\begin{array}{c} V_{0}\left(S\right)=\left\{\begin{array}{cc} 0\text{,} & \lambda_{2}>0\text{,}\\ \exp\;\left(-\frac{R_{b}}{2\beta^{2}}\right)\left[1-\exp\;\left(-\frac{S^{2}}{2C^{2}}\right)\right]\text{,} & others\text{,} \end{array}\right. \end{array}$$

(5) $$\begin{array}{c} \left\{\begin{array}{c} R_{b}=\frac{\lambda_{1}}{\lambda_{2}}\text{,}\\ S={\left\|H\right\|}_{F}=\sqrt{\underset{j\leq 2}{\sum }\lambda_{j}^{2}}\text{,} \end{array}\right. \end{array}$$

*β* and *c* are used to adjust the sensitivity of *R*_*b*_ and *S*, which is generally set to 0.5 and 15.

## 3. Classic Computational Intelligence Algorithms for Crack Image

### 3.1. K-Means Clustering

The fundamental premise of the K-means clustering algorithm is to group data samples into k-classes, calculate the distance from other points to the center of the cluster, assign the samples that are geographically closest to the center of the cluster to a single class, calculate the new center of the cluster, and update the attribution of each sample iteration after iteration until the center of the cluster does not change significantly and satisfactory clustering results are obtained. The K-means clustering technique is a part of the hard clustering division. Within this division, the Euclidean distance is utilized as the similarity measure, and the sum of squares of errors is utilized as the criterion function to calculate the cost value of repetitive operations. The total number of deviations that exist between the sample and the cluster center is referred to as the sum of squared errors. When determining the accuracy of a cluster, the greater the cost value, the more significant the mistake. The optimal partition can be obtained only in the case where the cost function is the smallest possible value.

Assuming that the data samples are a data se t*X*={*x*_1_, *x*_2_, ⋯, *x*_*n*_} with *n* samples, the *n* samples will eventually be divided into *k* clusters, and the *k* clusters meet the following conditions: (1) each classification cannot be empty; (2) each data sample exists in only one classification. First, *k* samples are randomly selected as the initial cluster centers of K clusters, where Euclidean distance is used as the similarity measurement and each sample is classified into the category of the nearest cluster center according to the size of the distance. The average value of all data samples in each cluster is calculated with Equation (6) as the new cluster center:

(6) $$\begin{array}{c} m_{i}=\frac{1}{N_{i}}\sum_{j=1}^{N_{i}}x_{ij},i=1,2,\cdots ,k\text{,} \end{array}$$

where *N*_*i*_ is the number of samples in the *i*th cluster.

The similarity between each sample and the new cluster center is recalculated, and the samples are reclassified before calculating the new cluster center. If the cluster center moves little or no samples are reassigned before and after clustering, the sample's clustering iteration terminates and the clustering cost function reaches a minimum. The following is the equation for the cost function of K-means clustering:

(7) $$\begin{array}{c} J={\sum_{i=1}^{k}\sum_{j=1}^{N_{i}}\left\|x_{ij}-m_{i}\right\|}_{2}^{2}\text{.} \end{array}$$

In the process of each iteration, judging whether each sample is assigned to the correct class is the key of K-means. If the classification is incorrect, it is necessary to make changes. After all samples are classified and corrected, it is judged that all samples are classified into the correct class, and then the cluster center is calculated and modified, and the next iteration is started. The steps of K-means clustering algorithm are described as follows.

**Step 1 .**

Divide the samples into *k* groups.

**Step 2 .**

Randomly select *k* points as the cluster center (*m*_1_, *m*_2_, ⋯, *m*_*k*_) of *k* groups.

**Step 3 .**

Calculate the distance from each sample *x*_*i*_ to *k* cluster centers (*m*_1_, *m*_2_, ⋯, *m*_*k*_).

**Step 4 .**

For each sample *x*_*i*_, assign it to the cluster with the closest center.

**Step 5 .**

Calculate the new cluster center according to Equation (5).

**Step 6 .**

Calculate the cost function according to Equation (6).

**Step 7 .**

If the value of the cost function *J* converges, output the cluster centers (*m*_1_, *m*_2_, ⋯, *m*_*k*_) and the algorithm terminates. Otherwise, return to Step 2.

### 3.2. Fuzzy C-Means

In FCM algorithm, it is given that the data set *X*={*x*_1_, *x*_2_, ⋯, *x*_*n*_} with *n* samples has divided the samples into *c* fuzzy groups, where 2 ≤ *c* ≤ *n* is the clustering centers of *c* fuzzy groups. The Euclidean distance function is used to calculate the similarity between the samples and the clustering centers, assign membership values to them, obtain the membership matrix U of all samples, and then obtain a new clustering center until the objective function representing the dissimilarity index finally reaches the minimum value. Since the membership matrix U has a normalization regulation, the total membership degree of a sample for each category is equal to 1.

(8) $$\begin{array}{c} \sum_{i=1}^{c}U_{ij}=1,\forall j=1,2,\cdots ,n\text{.} \end{array}$$

Therefore, the objective function of FCM can be described as follows:

(9) $$\begin{array}{c} J\left(U,c_{1},c_{2},\cdots ,c_{c}\right)=\sum_{i=1}^{c}\sum_{j=1}^{n}u_{ij}^{m}d_{ij}^{2}\text{.} \end{array}$$

When the objective function *J*(*U*, *c*_1_, *c*_2_, ⋯, *c*_*c*_) takes the minimum value, the best clustering effect can be obtained.

(10) $$\begin{array}{c} \hat{J}\left(U,c_{1},c_{2},\cdots ,c_{c}，\lambda_{1}，\cdots ，\lambda_{n}\right)\\ =\sum_{i=1}^{c}\sum_{j=1}^{n}u_{ij}^{m}d_{ij}^{2}+\sum_{j=1}^{n}\lambda_{i}\left(\sum_{i=1}^{c}u_{ij}-1\right)\text{,} \end{array}$$

where *λ*_*j*_(*j*=1, ⋯, *n*) is the Lagrangian multiplier of *n* constraint expressions in (9). The obtained value when the derivative is 0 is the value that minimizes the (10), so the necessary conditions are as follows:

(11) $$\begin{array}{c} c_{i}=\sum_{j=1}^{n}\frac{u_{ij}^{m}x_{j}}{\sum_{j=1}^{n}u_{ij}^{m}}\text{,}\\ u_{ij}=\frac{1}{\sum_{k=1}^{c}\left(d_{ij}\right./d_{kj}{\text{)}}^{\left(2/m-1\right)}}\text{.} \end{array}$$

The above two essential requirements yield the new cluster center and membership matrix. The fuzzy C-means clustering algorithm is a straightforward iterative procedure.

### 3.3. Maximum Entropy Clustering

The maximum entropy clustering algorithm is a deterministic annealing clustering algorithm based on the entropy. By taking the entropy as a constraint, it tries to maximize the entropy while minimizing the fuzzy distortion. The algorithm regards the entropy function as a component of the objective function to achieve a better division of the data set.

The maximal entropy clustering algorithm can be described by the following mathematical model. Given *n* vector *X*={*x*_1_, *x*_2_, ⋯, *x*_*n*_} ∈ *R*^*s*^ with *s*-dimensional space, it is assumed that there are *c* fuzzy class according to some similarity measures. The center of the *i*th fuzzy class is expressed as *y*_*i*_, *i*=1,2, ⋯, *c*, and the posterior probability of each vector belonging to the fuzzy class is represented by a set of real numbers between 0 and 1, which can be denoted as {P=(*py*_*i*_|*x*_*i*_)} ∈ *R*^*cn*^(*i*=1, ⋯, *c*; *k*=1, ⋯, *n*. This set of real numbers satisfies the following constraints: ∑_*i*=1_^*c*^*p*(*y*_*i*_|*x*_*k*_)=1; *p*(*y*_*i*_|*x*_*k*_) ∈ [0,1]; and ∑_*i*=1_^*c*^*p*(*y*_*i*_|*x*_*k*_) > 0. Therefore, the objective function of the maximum entropy clustering algorithm is defined as

(12) $$\begin{array}{c} J=\overset{c}{\underset{i=1}{\sum }}\overset{n}{\underset{k=1}{\sum }}p\left(y_{i}\left|x_{k}\right.\right){\left(x_{k}-y_{i}\right)}^{2}+T\overset{c}{\underset{i=1}{\sum }}\overset{n}{\underset{k=1}{\sum }}p\left(y_{i}\left|x_{k}\right.\right)\log\;p\left(y_{i}\left|x_{k}\right.\right), \end{array}$$

where *Y*=(*y*_1_, *y*_2_, ⋯,*y*_*c*_)^*T*^ ∈ *R*^*CS*^; *d*(*x*_*k*_, *y*_*i*_)=‖*x*_*k*_ − *y*_*i*_‖^2^. The maximum entropy clustering algorithm is to find a probability distribution and a set of cluster centers to make the fuzzy distortion degree *L* the smallest and the entropy *H* to be the largest. Generally, the Lagrange multiplier method can be used to obtain the minimum value (*p*^*∗*^, *y*^*∗*^) of *J*.

It can be seen from the description of the maximum entropy clustering algorithm that when *T* is large, ∑_*i*=1_^*c*^∑_*k*=1_^*n*^*p*(*y*_*i*_|*x*_*k*_)log *p*(*y*_*i*_|*x*_*k*_) plays a major role in the objective function. In addition, the probability of each sample belonging to *c* cluster centers is equal, which is approximately equal to 1/*c*. As *T* gradually decreases, ∑_*i*=1_^*c*^∑_*k*=1_^*n*^*p*(*y*_*i*_|*x*_*k*_)(*x*_*k*_ − *y*_*i*_)^2^ plays a more and more important role in the objective function. In addition, the probability of each sample belonging to the cluster center closest to it is increasing. When *T* = 0, each sample belongs to the nearest cluster center with probability 1.

### 3.4. Gaussian Mixture Model

Gaussian mixture models are about quantifying things precisely with multiple Gaussian probability density functions. Each Gaussian mixture model is composed of *N* Gaussian distributions, each of which is called a cluster. These Gaussian distributions are combined to form the probability density function of the Gaussian mixture model.

(13) $$\begin{array}{c} p\left(x\right)=\sum_{n=1}^{N}p\left(n\right)p\left(X\left|n\right.\right)\text{,}\\ p\left(x\right)=\sum_{n=1}^{N}\pi_{n}N\left(x\left|u_{n}\right.,\Sigma_{n}\right)\text{,} \end{array}$$

where *N* is the number of models; *π*_*n*_ represents the weight coefficient, which means the probability that each cluster class is selected, and ∑_*n*=1_^*N*^*π*_*n*_=1; and *N*(*x*|*u*_*n*_, Σ_*n*_) is the Gaussian distribution density, Σ_*n*_=*δ*_*n*_^2^. *δ*_*n*_^2^ represents the standard deviation of the *n*th class, so the *n*th Gaussian model is denoted as follows:

(14) $$\begin{array}{c} N\left(x\left|\mu_{n},\delta_{n}^{2}\right.\right)=\frac{1}{\sqrt{2\pi }\delta_{n}}\exp\;\left(-\frac{{\left(x-\mu_{n}\right)}^{2}}{2\delta_{n}^{2}}\right)\text{.} \end{array}$$

Assuming that there are K collected samples, these samples can be considered to obey a Gaussian distribution, and then the likelihood function of GMM can be written as

(15) $$\begin{array}{c} \overset{K}{\underset{i=1}{\prod }}p\left(x\right)=\sum_{i=1}^{K}\log\;\left(\sum_{n=1}^{N}\pi_{n}N\left(x\left|\mu_{n},\delta_{n}^{2}\right.\right)\right)\text{.} \end{array}$$

Since the maximum value cannot be obtained directly in (14), so the EM algorithm must be adopted. The steps of EM algorithm for parameter estimation of Gaussian mixture model are described as follows:

**Step 8 .**

Determine initial value *π*, *μ*, *δ*.

**Step 9 .**

Calculate the probability that the sample is Gaussian distribution.

(16) $$\begin{array}{c} \gamma \left(i,n\right)=\frac{\pi_{n}N\left(x_{i}\left|\mu_{i},\delta_{i}^{2}\right.\right)}{\sum_{j=1}^{N}\pi_{j}N\left(x_{i}\left|\mu_{j},\delta_{j}^{2}\right.\right)}\text{.} \end{array}$$

**Step 10 .**

Calculate maximum likelihood function of *μ*_*n*_ and *δ*_*n*_^2^.

(17) $$\begin{array}{c} u_{n}=\frac{1}{K_{n}}\sum_{i=1}^{K}\gamma \left(i,n\right)x_{i}\text{,}\\ \delta_{n}^{2}=\frac{1}{K_{n}}{\sum_{i=1}^{K}\gamma \left(i,n\right)\left(x_{i}-\mu_{n}\right)\left(x_{i}-\mu_{n}\right)}^{T}\text{.} \end{array}$$

**Step 11 .**

Calculate maximum likelihood function of *π*_*n*_, namely, *π*_*n*_=*K*_*n*_/*K*.

**Step 12 .**

Repeat iteration steps 2–4 until the parameter likelihood function value converges.

### 3.5. Mean-Shift Clustering

Mean-shift is a prediction method of gradient search through nonparametric probability density estimation, which classifies the sample data and counts the pattern categories in the feature space of the data samples. The mean-shift-based image segmentation is to first estimate the probability density, then find the convergence point through gradient search, use the convergence point to filter the image, and finally achieve image segmentation.

Nonparametric estimation refers to the process of estimating the density function using sample data sets. Kernel density estimation is the most commonly used nonparametric estimation, which is the process of processing the sample according to the kernel function *K*(*x*) to obtain the density function. For a Euclidean space with *d*-dimensional, *x* is one of the eigenvectors, and R is a real number field. If a function *K* : *R*^*d*^⟶*R* follows *K* : [0, +*∞*]⟶*R*, we can obtain *K*(*x*)=*C*_*k*_*k*(‖*x*‖^2^), where the modulus of *x* is denoted as ‖*x*‖^2^=*x*^T^*x*; *C*_*k*_ is a standardized constant; and *k* obeys non-negative and piece-wise continuous and ∫*k*(*r*)*dr* < *∞*.

Given the known kernel function *K*(*x*) and bandwidth matrix *H*_*i*_(*x*) of *n* sampling points {*x*_*i*_, 1 ≤ i ≤ n} in the space *R*^*d*^, the kernel density estimation formula of the density function can be denoted as

(18) $$\begin{array}{c} \hat{f}\left(x\right)=\sum_{i=1}^{n}c_{k}\omega_{i}{\left|H_{i}\right|}^{-1/2}K\left({\left\|x-x_{i}\right\|}^{2}\left|H_{i}\right|\right)\text{,} \end{array}$$

*w*(*x*_*i*_) ≥ 0 is the weight of the sampling point, which satisfies ∑*w*(*x*_*i*_)=1 and abbreviates as *ω*_*i*_. The kernel function *K*(*x*) is the similarity measurement between the sample *x*_*i*_ and the center *x*. The bandwidth matrix *H*_*i*_ represents the range of the kernel function estimation. The density function estimate $\hat{f}\left(x\right)$ is obtained by the weighted sum of the kernel function at each sample.

Generally, the density at *x* is always smaller than that at *m*_*H*_*i*__(*x*), and the mean-shift vector should move in the direction of high density. The convergence point of the mean-shift algorithm is the local density maximum point. In order to describe the mean-shift algorithm more simply, it is given that the weights between sampling points are equal, namely, *ω*(*x*_*i*_)=1/*n*. Since the bandwidth matrix is proportional to the identity matrix *H*_*i*_=*h*^2^*I*, then the mean-shift iteration formula can be written as follows:

(19) $$\begin{array}{c} m_{h}\left(x\right)=\frac{\sum_{i=1}^{n}g\left({\left\|\left(x-x_{i}\right)h^{-1}\right\|}^{2}\right)x_{i}}{\sum_{i=1}^{n}g\left({\left\|\left(x-x_{i}\right)h^{-1}\right\|}^{2}\right)}\text{.} \end{array}$$

The mean-shift algorithm firstly and randomly selects a search area circle in the sample and calculates the average value of all sample points in the search area through the iterative formula. The density of the newly obtained mean point must be greater than the density at the initial center point. Repeat the above steps until the density change is less than a certain value, and then converge to the maximum density point.

### 3.6. Hierarchical Clustering

In the method known as hierarchical clustering, a structure that looks like a tree is built to correlate with the samples that are going to be clustered. This is done in order to facilitate the clustering process. The process of hierarchical clustering can be further subdivided into a variety of distinct clustering algorithms depending on the direction in which the hierarchy is constructed, either from the bottom up or from the top down. These distinct algorithms can then be used to cluster data in a variety of different ways. Because of the flexibility of the hierarchical structure, it is possible to make this distinction in either direction during its construction. When applying the bottom-up methodology, the first thing that needs to be done is to treat every single sample as if it were its own cluster. After that, it combines these atomic clusters to produce clusters that are progressively larger until either all of the samples are included within a single cluster or a termination condition is satisfied, whichever occurs first. After that, it moves on to the next step. The concept of similarity between clusters can be understood quite differently depending on the approach that is taken, despite the fact that the vast majority of hierarchical clustering algorithms fall into this category. Hierarchical clustering takes a different approach, one that works from the bottom up, as opposed to the strategy that operates in the opposite direction, from the top down. It starts by grouping all of the samples together into a cluster, and then it gradually divides that larger cluster into smaller and smaller clusters until either every object spontaneously forms its own cluster or it satisfies a condition that causes it to stop.

The relative connectivity between two clusters *c*_*i*_ and*c*_*j*_ is defined as the absolute connectivity between two clusters *c*_*i*_ and *c*_*j*_ divided by two relative connectivities in *c*_*i*_ and *c*_*j*_.

(20) $$\begin{array}{c} RI\left(c_{i},c_{j}\right)=\frac{\left|{EC}_{\left(c_{i},c_{j}\right)}\right|}{\left(\left|{EC}_{\left(c_{i}\right)}\right|+\left|{EC}_{\left(c_{j}\right)}\right|\right)/2}\text{,} \end{array}$$

where |EC_(*c*_*i*_, *c*_*j*_)_| is the edge-out containing *c*_*i*_ and *c*_*j*_ clusters, so that the cluster can be decomposed into |EC_(*c*_*i*_)_| and |EC_(*c*_*j*_)_|. The relative approach degree between two clusters *c*_*i*_ and *c*_*j*_ is the absolute approach degree between two clusters *c*_*i*_ and *c*_*j*_ divided by the approach degree within two clusters *c*_*i*_ and *c*_*j*_:

(21) $$\begin{array}{c} RC\left(c_{i},c_{j}\right)=\frac{S_{EC\left(c_{i},c_{j}\right)}}{\left(\alpha_{i}S_{EC\left(c_{i}\right)}+\alpha_{j}S_{EC\left(c_{j}\right)}\right.}\text{.} \end{array}$$

## 4. Experiment

### 4.1. Evaluation Indicators

In order to provide a reasonable evaluation of the clustering performance of each clustering algorithm, this study uses the NMI and the RI evaluation indicators to analyze the performance of each comparison algorithm. These two indicators have a value range of [0, 1], and a good rule of thumb to follow is that a higher number indicates a higher level of clustering performance. Calculating the degree of error that occurred during the process of judging the results of the fracture segmentation can be done with the help of an index known as the NMI index. When this value is increased, the crack segmentation will become more accurate as a result. The magnitude of the value is inversely proportional to the extent of this improvement. The RI index examines and quantifies the degree to which the segmentation results of the crack segmentation algorithm and the results of the ground-truth segmentation are consistent along their edges. This is accomplished by comparing the segmentation results of the two sets of data. This is accomplished by contrasting the outcomes of the two studies with one another. When the value is increased, the algorithm will produce segmentation results that are of a higher quality than they were before.

These cluster methods are first tested on a Windows computer equipped with a 2.50 GHz Intel Core i5-1135G7 processor and 4 gigabytes of RAM, and then they are implemented in MATLAB using the MATLAB R2012b software, which is run on a Windows 10 64-bit operating system. Finally, the cluster methods are validated on a Windows computer.

### 4.2. Data Sources

**Figure 3:**
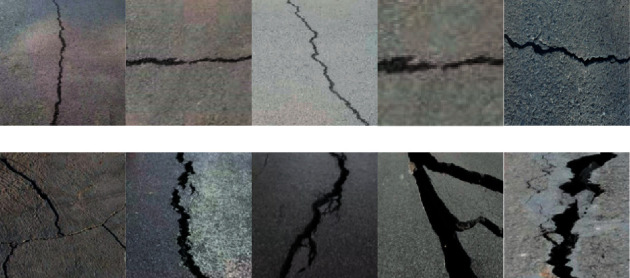
The samples of typical crack images.

According to the types and characteristics of pavement cracks, this paper selects some images of different locations, different environments, and different time periods. These samples cover typical cracks such as bifurcation, uneven thickness, uneven illumination, occlusion, road markings, and their shadows, which can meet the testing requirements of crack image clustering algorithm. These crack images can be found and downloaded from the Internet, such as https://img.xianjichina.com/editer/20210113/image/e80456e50e92c5eb0b8b03b.jpg. For testing requirements, the crack data set with a size of 128*∗*128 or 64*∗*64 pixels is formed by cutting out the crack images. Many of them suffer from the problems of occlusion and shadow interference cracks and fine cracks and carrying a large number of peripheral cracks, which is difficult to segment.  Figure 3 shows the typical crack images selected in this paper, the first row is single-crack images, and the second row is cracks with complex background interference. We manually annotate the ground-truth crack on test images for objective performance evaluation.

### 4.3. Experimental Analysis

In this article, several pavement crack images are processed with the help of six different clustering algorithms. The degree of complexity of the crack image has a significant bearing on the segmentation results achieved by the clustering algorithm. Additionally, because the acquisition equipment is susceptible to the influence of light intensity and noise, the brightness of various cracks in the crack image is typically uneven. This will have a negative impact on the effectiveness of the crack image segmentation. In order to properly segment cracks in an image, it is necessary to preprocess the image first.

In this study, an image enhancement algorithm was applied during the image preprocessing stage in order to get rid of any noise and interference that might have been present. Image enhancement will, simultaneously, improve the response value of crack image similarity measure, which will be helpful in improving the performance of crack segmentation. Even though the main body of the crack has been preserved thanks to image enhancement, the fine cracks and peripheral cracks have been lost in a significant way, which has resulted in the crack having poor continuity. In addition, the phenomenon of under-segmentation is more serious, and the segmentation itself presents a fracture phenomenon. The noise in the crack segmentation result has been greatly reduced, but the segmentation result makes it easy to lose details; the smaller crack area is easy to connect, but it is easy to distinguish the subtle and peripheral cracks as noncrack area, and the cracks are disconnected, which results in serious over-segmentation of the cracks. Therefore, the preprocessing method that was used in this paper helps retain the details, and the results of this method do not affect how well the six different clustering algorithms perform when compared to one another.

**Figure 4:**
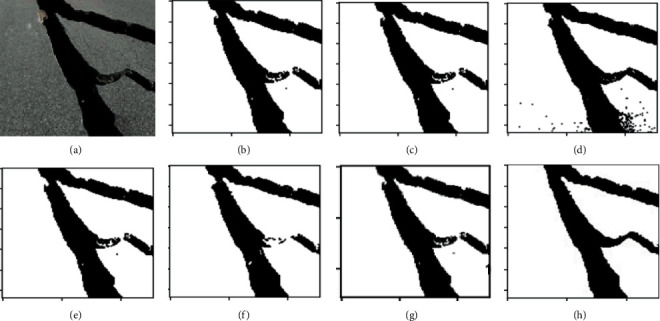
Performance comparison for different cluster algorithms. (a) RAW images; (b) K-means; (c) FCM; (d) MEC; (e) GMM; (f) mean-shift; (g) HC; (h) ground-truth.

**Figure 5:**
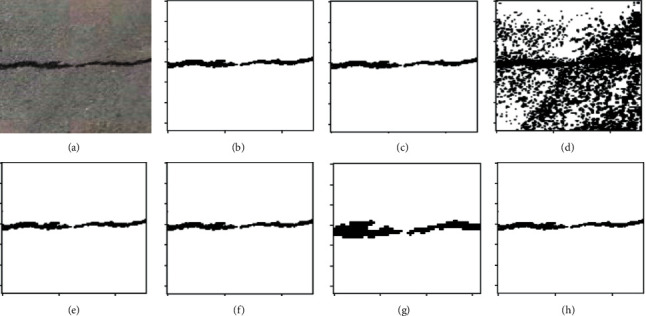
Performance comparison for different cluster algorithms. (a) RAW images; (b) K-means; (c) FCM; (d) MEC; (e) GMM; (f) mean-shift; (g) HC; (h) ground-truth.

**Figure 6:**
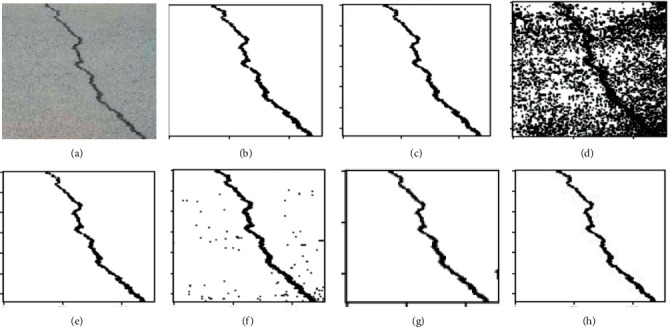
Performance comparison for different cluster algorithms. (a) RAW images; (b) K-means; (c) FCM; (d) MEC; (e) GMM; (f) mean-shift; (g) HC; (h) ground-truth.

K-means clustering (K-means), fuzzy c-means clustering (FCM), maximum entropy clustering (MEC), Gaussian mixture mode (GMM), mean-shift, and hierarchical clustering (HC) are used as comparison clustering algorithms in order to verify the comprehensive segmentation performance of these clustering algorithms. Test samples are randomly selected from the pavement crack database. Because of the constraints imposed by the available space, we can only select a select few examples of typical image segmentation results for analysis, as can be seen in Figures 4–6.

It can be seen from the segmentation results that different algorithms can basically achieve crack segmentation, but the segmentation results show obvious differences. The K-means algorithm is highly sensitive to outlier noise, and the selection of cluster centers directly affects the segmentation results. The segmentation results only preserve the main structure of the crack. Noise and the interference of the complex background cause the segmentation algorithm to lose a lot of details, especially the edge information.

The segmentation results show a fracture phenomenon, there is a lot of noise, and there is an over-segmentation phenomenon such as judging the complex background as a crack area. The neighborhood information that introduces crack pixels into the feature is improved compared with the segmentation result of the K-means algorithm, but the noise sensitivity is still high, which easily leads to the islanding effect. It is easy to judge the background as a crack at the tip and subtle parts of the image, and there is still a lot of noise in the noncrack area.

Based on the gray distribution characteristics of cracks, the GMM algorithm uses Gaussian distribution to fit the feature distribution of cracks and completes the segmentation of crack images through adaptive thresholds, but the selection of parameter values calculated by the EM algorithm directly affects the segmentation results. Although the segmented cracks are relatively continuous, it is easy to mistakenly identify noncrack areas as crack area, showing an over-segmentation phenomenon, and there are a small number of noise points around the cracks, as shown in Figure 5(d). In a single crack, it is easy to misjudge the crack target as a noncrack with under-segmentation phenomenon.  Figure 6(g) shows the segmentation result of the algorithm in this paper. The segmentation effect is significantly improved, and the crack fracture phenomenon is improved. The mean-shift algorithm in this paper can completely segment the crack structure and effectively solve the detailed problems of crack image segmentation, such as fine cracks, peripheral cracks, and branch cracks. At the same time, the hierarchical clustering algorithm can extract clear crack area, which can well overcome the segmentation difficulties caused by uneven gray scale, noise, and overlapping edge. The crack segmentation results can provide high-quality reference for subsequent data analysis and crack classification.

**Figure 7:**
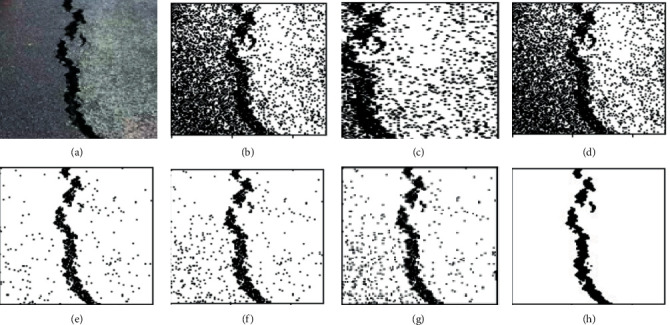
Performance comparison for different cluster algorithms with noise ((zero mean and 0.01 variance)). (a) RAW images; (b) K-means; (c) FCM; (d) MEC; (e) GMM; (f) mean-shift; (g) HC; (h) ground-truth.

Since this paper mainly analyzes the processing effects of different clustering algorithms on different noisy images, Figure 7 shows the result of adding Gaussian noise with zero mean and 0.01 variance to the raw image and clustering directly. It can be seen that the introduction of noise significantly changes the segmentation performance. Noise interference appeared in all results, and some background areas were mistaken for cracks. Although the image enhancement function is added in the preprocessing stage of this paper, there are still some interferences, resulting in errors in the results, but the introduction of preprocessing is also significantly better than the results of direct clustering. It is worth noting that although all comparison algorithms have some scattered speckle areas disturbed by noise, the interference can be eliminated by edge detection.

The statistics in Tables 1 and 2 show the quantitative indicators of different clustering algorithms for the same crack image with/without noise. In Table 1, the NMI index of mean-shift algorithm reaches 82.86%, which is 8.83%, 2.06%, and 3.85% higher than that of K-means algorithm, MEC algorithm, and FCM algorithm, respectively; as for the RI index in Table 2, K-means algorithm, MEC algorithm, and FCM algorithm are 9.52%, 6.27%, and 1.23% higher, respectively. Therefore, for pavement crack cluster application, the measurement index of mean-shift algorithm is better than the comparison algorithm and meets the needs of engineering detection.

According to the NMI-mean comparison of each clustering algorithm, the effect of MEC is the worst, the effect of other algorithms is not bad, and the gap is small, where the mean-shift clustering algorithm has the best effect. Various clustering algorithms have been adjusted and optimized to achieve the optimal segmentation effect. In this experiment, it seems that the NMI-based evaluation results are more in line with the performance of segmentation effects, while the segmentation results that seem to have poor effects can also have RI value above 0.5. The segmentation effect of these cluster algorithms is significantly worse after adding Gaussian noise. According to the NMI value, the mean-shift clustering algorithm has the best de-noise effect, while the performance of K-means algorithm and FCM algorithm decreases significantly after adding noise.

## 5. Conclusion

The purpose of this study is to evaluate and analyze the performance of complex crack images in a variety of different environments using a number of different standard clustering algorithms. NMI and RI have been selected to serve as assessment indices for the purpose of determining whether or not the comparison algorithm is effective. The performance of a comparison analysis on the noisy photos is an additional component of the experiment that needs to be carried out. The NMI value indicates that the mean-shift clustering algorithm has the best de-noise effect, whereas the performance of the K-means algorithm and the FCM algorithm significantly decreases after noise is added to the data. The findings of the experiments indicate that the addition of Gaussian noise makes these cluster algorithms significantly less effective at segmenting the data than they were before.

Despite the fact that many different clustering algorithms have been proposed and are continually being improved, there has not been developed a single algorithm that is appropriate for a variety of data features. This is primarily the result of the fact that the algorithms used for clustering put an excessive amount of emphasis on the compactness that exists within clusters as well as the differences that exist between clusters. The arrival of the era of big data has resulted not only in an increase in the total amount of data, but also in an increase in the complexity of the data structure. This is due to the fact that the total amount of data has increased. Finding a way to build a clustering algorithm and evaluation index that is flexible enough to accommodate a wide range of different scenarios will be an essential goal for the work that will be done in the future.

## Figures and Tables

**Table 1:** Performance comparison of different clustering algorithms for the same crack image without noise.

Images	Indexes	Algorithms					
		K-means	FCM	MEC	GMM	HC	Mean-shift
1	RI	0.9637	0.9639	0.9358	0.9637	0.9720	0.9639
	NMI	0.8371	0.8379	0.7707	0.8366	0.8792	0.8379

2	RI	0.9985	0.9985	0.5521	0.9973	0.9979	0.9985
	NMI	0.9621	0.9621	0.0792	0.9402	0.9517	0.9630

3	RI	0.9980	0.9996	0.5286	0.9944	0.9756	0.9995
	NMI	0.9516	0.9886	0.0624	0.8821	0.6861	0.9853

4	RI	0.9650	0.9664	0.6653	0.9655	0.9655	0.9659
	NMI	0.7014	0.7082	0.2110	0.7079	0.7090	0.7101

5	RI	0.9374	0.9383	0.8837	0.9381	0.9300	0.9386
	NMI	0.7311	0.7379	0.5630	0.7371	0.6902	0.7448

6	RI	0.5918	0.5933	0.5192	0.9885	0.9837	0.9867
	NMI	0.0669	0.0673	0.0442	0.7092	0.6192	0.6842

7	RI	0.9615	0.9606	0.5342	0.9696	0.9681	0.9702
	NMI	0.5424	0.5377	0.0585	0.6143	0.6015	0.6194

8	RI	0.9880	0.9880	0.5187	0.9916	0.9919	0.9922
	NMI	0.8694	0.8694	0.1042	0.8941	0.8989	0.9018

9	RI	0.9874	0.9874	0.5338	0.9843	0.9857	0.9956
	NMI	0.8827	0.8827	0.1383	0.8668	0.8718	0.9495

Average	RI-mean	0.9324	0.9329	0.6302	0.9770	0.9745	0.9790
	RI-std	0.1219	0.1216	0.1558	0.0182	0.0188	0.0195
	NMI-mean	0.7272	0.7324	0.2257	0.7987	0.7675	0.8218
	NMI-std	0.2652	0.2691	0.2455	0.1034	0.1248	0.1282

**Table 2:** Performance comparison of different clustering algorithms for the same crack image with noise (zero mean and 0.01 variance).

Images	Indexes	Algorithms					
		K-means	FCM	MEC	GMM	HC	Mean-shift
1	RI	0.8358	0.8358	0.8358	0.8513	0.8508	0.8633
	NMI	0.4663	0.4663	0.4663	0.5134	0.5116	0.5555

2	RI	0.5230	0.5177	0.5177	0.7176	0.8753	0.9565
	NMI	0.0620	0.0600	0.0600	0.1379	0.2645	0.4370

3	RI	0.5104	0.5065	0.5065	0.5486	0.9542	0.6283
	NMI	0.0367	0.0350	0.0350	0.0450	0.1907	0.0817

4	RI	0.5653	0.5653	0.5648	0.9200	0.8567	0.9049
	NMI	0.1035	0.1035	0.0992	0.4207	0.3250	0.3901

5	RI	0.8528	0.8528	0.8528	0.8580	0.8506	0.8578
	NMI	0.4496	0.4496	0.4496	0.4759	0.4422	0.4741

6	RI	0.5063	0.5039	0.5035	0.5533	0.9703	0.9569
	NMI	0.0297	0.0289	0.0169	0.0403	0.3236	0.2967

7	RI	0.5172	0.5134	0.5006	0.6674	0.9459	0.8982
	NMI	0.0267	0.0252	0.0022	0.0688	0.3584	0.2318

8	RI	0.5957	0.5885	0.5885	0.9532	0.9345	0.9233
	NMI	0.1124	0.1083	0.1083	0.5882	0.5234	0.4900

9	RI	0.5366	0.5432	0.5130	0.7466	0.6992	0.7747
	NMI	0.0952	0.0996	0.1155	0.2266	0.1921	0.2704

Average	RI-mean	0.6048	0.6030	0.5981	0.7573	0.8820	0.8627
	RI-std	0.1309	0.1318	0.1346	0.1409	0.0788	0.0984
	NMI-mean	0.1536	0.1529	0.1503	0.2796	0.3479	0.3586
	NMI-std	0.1655	0.1659	0.1687	0.2076	0.1172	0.1418

## Data Availability

The data set used to support the findings of this study is available from the corresponding author upon request.
